# Similar expression profiles in CD34^+^ cells from chronic phase chronic myeloid leukemia patients with and without deep molecular responses to nilotinib

**DOI:** 10.18632/oncotarget.24954

**Published:** 2018-04-03

**Authors:** Ami B. Patel, Thoralf Lange, Anthony D. Pomicter, Christopher J. Conley, Christina A. Harrington, Kimberly R. Reynolds, Todd W. Kelley, Thomas O’Hare, Michael W. Deininger

**Affiliations:** ^1^ Division of Hematology and Hematologic Malignancies, The University of Utah, Salt Lake City, UT, USA; ^2^ Huntsman Cancer Institute, The University of Utah, Salt Lake City, UT, USA; ^3^ Department of Pathology, The University of Utah, Salt Lake City, UT, USA; ^4^ Integrated Genomics Laboratory, Oregon Health and Science University, Portland, OR, USA; ^5^ University of Leipzig, Division of Haematology and Oncology, Leipzig, Germany

**Keywords:** chronic myeloid leukemia, BCR-ABL1, deep molecular response, treatment-free remission

## Abstract

The life expectancy of patients with chronic phase chronic myeloid leukemia on tyrosine kinase inhibitor therapy now approaches that of the general population. Approximately 60% of patients treated with second generation tyrosine kinase inhibitors achieve a deep molecular response, the prerequisite for a trial of treatment-free remission. Those patients unlikely to achieve deep molecular response may benefit from more intensive therapy up front. To identify biomarkers predicting deep molecular response we performed transcriptional profiling on CD34^+^ progenitor cells from newly diagnosed chronic phase chronic myeloid leukemia patients treated with nilotinib on a prospective clinical trial. Using unsupervised and targeted analytical strategies, we show that gene expression profiles are similar in patients with and without subsequent deep molecular response. This result is in contrast to the distinct expression signature of CD34^+^ chronic phase chronic myeloid leukemia patients failing to achieve a cytogenetic response on imatinib and suggests that deep molecular response to second-generation tyrosine kinase inhibitors is governed by the biology of more primitive chronic myeloid leukemia cells or extrinsic factors.

## INTRODUCTION

Most chronic phase chronic myeloid leukemia (CP-CML) patients treated with tyrosine kinase inhibitors (TKIs) have excellent outcomes, with overall survival driven primarily by co-morbidities [1]. Although TKIs are generally well tolerated, long-term use can lead to complications. Imatinib has an excellent safety record but multiple persistent low grade side effects are common. The second generation (2G) TKIs nilotinib and dasatinib exhibit improved tolerance, yet are associated with potentially severe cardiovascular and/or pulmonary toxicity [2, 3]. Accordingly, therapeutic goals in CML have shifted from overall survival to treatment free remission (TFR), defined as the ability to maintain major molecular response (MMR) after TKI discontinuation. Current guidelines recommend stable deep molecular response (DMR; reduction of BCR-ABL1 transcripts by ≥4-log) as the minimum prerequisite for a trial of TFR [4, 5]. In the DASISION and ENESTnd trials, DMR rates by 5 years were approximately 40% on imatinib and 60% on 2G TKIs [6, 7].

Efforts to improve rates of DMR have focused on switching patients on imatinib who plateau above DMR to a 2G TKI, reflecting the lack of biomarkers to predict DMR up-front. We previously reported a 75-probe gene expression signature derived from CD34^+^ cells of imatinib-naïve CP-CML patients that predicts major cytogenetic response (MCyR; <36% Ph^+^ metaphases) to imatinib [8]. We hypothesized a similar approach may allow for prediction of DMR and performed transcriptional profiling on CD34^+^ cells from newly diagnosed CP-CML patients prior to nilotinib treatment.

## RESULTS

**Figure 1:**
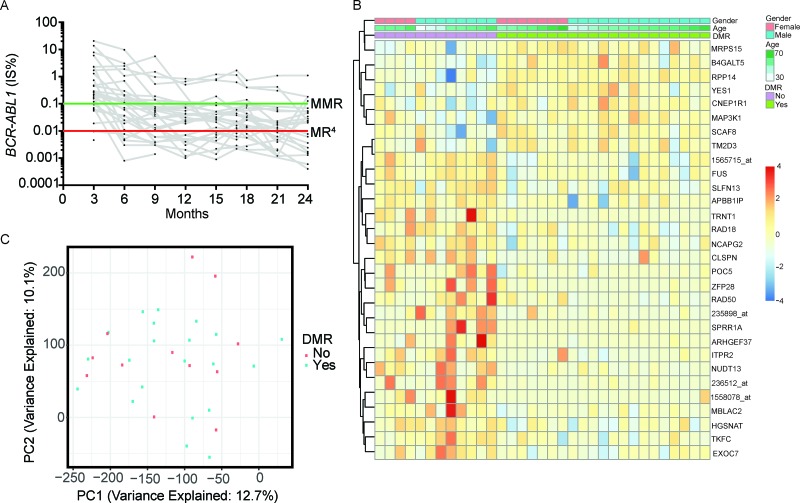
(**A**) Patient BCR-ABL1 qPCR results. (**B**) Heat map of the top 30 differentially expressed probes. (**C**) Principal component analysis of patient gene expression profiles.

Over 24 months, 95% (36/38) of patients achieved CCyR, 87% (33/38) achieved ≥MMR, and 58% (22/38) attained ≥MR4 (Figure 1A). These responses are comparable to ENESTnd results with respect to cumulative incidence of CCyR (87%) and MMR (71%) at 24 months and percentage of patients (44%) achieving ≥MR^4^ at any time [9].

Of 35 patients with high quality microarray data, two patients were excluded from downstream analysis due to insufficient qPCR monitoring. Of the remaining 33 patients, 21 were responders and 12 were non-responders. We compared the proportion of missing qPCR assessments between responders and non-responders. More data points were missing in the 21 responders [Q1 (3, 6 and 9-month) 11.1%, Q2 (12, 15, and 17-month) 22.2%, Q3 (18, 21, and 24-month) 44.4%] compared to the 12 non-responders (Q1 0%, Q2 0%, Q3 13.9%). Thus, lack of DMR in non-responders was not due to reduced frequency of testing. However, achieving DMR may have led to reduced protocol adherence in responders.

Baseline clinical parameters, CP-CML risk scores and proportion of *BCR-ABL1*-positive cells in CD34^+^ cells subjected to microarray were comparable between responders and non-responders (Table 1, Supplementary Figure 1).

**Table 1:** Comparison of baseline parameters, responders vs non-responders

Baseline parameters at diagnosis	Responders, median (range)	Non-responders, median (range)	*p*-value (Responders vs. Non-responders)
Spleen size (cm below costal margin)	0 (0–4)	2 (0–15)	0.13
Platelet count (k/µL)	406 (170–1949)	324 (124–973)	0.54
Sokal score	0.84 (0.56–2.77)	0.91 (0.45–1.72)	0.97
Hasford score	994 (84–2255)	747 (124–1437)	1.00
Peripheral blood eosinophil %	3 (0–8)	3 (0–11)	0.92
Peripheral blood basophil %	3 (0–18)	3 (0–9)	0.31
Peripheral blood blast %	1 (0–9)	0.5 (0–23)	0.92
% BCR-ABL1+ CD34+ cells by FISH	89 (49–100)	91 (67–100)	0.70
Missed PCR timepoints (out of 9)	2 (0–8)	0 (0–7)	0.09

We evaluated 54,675 probes for differential expression between responders and non-responders. At a false discovery rate of 0.05 we found no significant differences in expression. A heat map of the top 30 probes ranked by *limma p*-value revealed limited expression differences between responders and non-responders (Figure 1B). We assessed distribution of *p*-values and found it to be uniform, consistent with high quality data (Supplementary Figure 2). We performed principal component analysis on the most variable probes determined by median absolute deviation (2,374/54,675 probes; 4.3%). There was no clear separation of responders and non-responders after projecting the samples onto the top four principal components (Figure 1C).

Since data from the unsupervised analysis failed to identify differences in global gene expression profiles of responders and non-responders, we investigated three pre-specified sets of non-overlapping probes with a potential role in governing molecular response to nilotinib: (i) a 75-probe set signature previously shown to predict major cytogenetic response in CP-CML patients treated with imatinib (Supplementary Figure 3); (ii) 50 probes corresponding to genes implicated in CML stem cell persistence identified by literature review (Supplementary Figure 4A); and (iii) 365 probes associated with Wnt/β-catenin signaling (KEGG ID hsa04310) based on our finding that β-catenin activation is a feature of primary cytogenetic resistance (Supplementary Figure 4B) [8]. None of these probe sets showed structured differences between responders and non-responders.

## DISCUSSION

As survival of CML patients approaches that of the general population, TFR is emerging as a new therapy goal [4, 5]. This shift reflects increased concern about long-term toxicity of TKIs, side effects and costs of life-long TKI therapy [10, 11]. A stable DMR is required to justify a trial of TFR, but most patients will never achieve this depth of response [12–18]. Although late intensification improves response in some patients, the optimal window for more potent TKIs or drug combinations may be immediately after diagnosis, before recalcitrant clones are selected [19]. Risk scores identify patients less likely to achieve DMR, but lack precision. Early molecular response (EMR; *BCR-ABL1* < 10% IS at three months) is strongly associated with subsequent DMR, but intervention based on EMR still allows considerable time for evolution of TKI resistant clones [20].

We previously identified a gene classifier that predicted cytogenetic response in CP-CML patients treated with imatinib [8]. Analysis of CD34^+^ cells was critical for identification of this resistance signature [8, 21]. Using a similar approach, we were unable to identify a gene expression signature that predicted DMR with nilotinib in our cohort. This finding held regardless of whether we approached the data in an unsupervised or hypothesis-driven fashion. Importantly, microarray analysis was identical to McWeeney *et al.* [8]. Although unlikely to have affected results, it is noteworthy that methods of RNA and cDNA amplification differed from prior [8]. One limitation of our study is the small sample size, and it remains possible that a signature would be detectable in a larger cohort. Another is that cytogenetic response, used in our previous study, is driven by elimination of progenitor cells, while DMR may be dependent on elimination of more primitive CD34^+^38^–^ CML cells. As such, a DMR signature may have been obscured by a dominant CD34^+^38^+^ population. The recent inclusion of CD26 as a putative leukemia stem cell marker in CML suggests that going forward, the CD34^+^/CD38^–^/CD26^+^ compartment may be most appropriate for identifying a resistance signature governing deep responses [22, 23]. Failure to achieve DMR on nilotinib may also be related to parameters unidentifiable by gene expression analysis, such as post-translational modifications or changes in immune surveillance [24–27]. Lastly, host factors may influence DMR, such as drug metabolism or adherence. Adherence to TKI therapy varies widely and is associated with response outcomes [28]. In summary, we have not been able to identify a gene expression profile that predicts DMR in CP-CML patients treated with nilotinib. Further research should focus on more primitive populations of leukemia cells and host factors.

## MATERIALS AND METHODS

### Patients

CAMN107AUS21T (NCT01061177) was a single-arm study testing the efficacy of nilotinib 300 mg twice daily in patients with newly diagnosed CP-CML. Details on the overall results have been published [29]. Patients in this substudy provided an additional consent and were followed for two years.

### Molecular monitoring

Peripheral blood BCR-ABL1 transcripts were quantified in a central lab (University of Leipzig, Germany). Results were normalized and expressed on the international scale (IS). Molecular response was classified according to current recommendations [30]. DMR was defined as achievement of a >4-log reduction in BCR-ABL1 transcript at any point during nilotinib therapy with responder vs non-responder cohorts denoted accordingly.

### Isolation of CD34^+^ cells

Blood collected prior to first dose of nilotinib was shipped to the University of Leipzig. CD34^+^ cells were isolated using immunomagnetic beads and cryopreserved in aliquots. CD34^+^ cells were shipped to The University of Utah and cells were thawed at 37° C, and incubated with CD34-APC (4H11, eBioscience, Thermo Fisher Scientific, Waltham, MA) and CD45-FITC (H130, BD Biosciences, San Jose, CA) monoclonal antibodies. Double positive cells were sorted with a BD FACSAria3 directly into RLT plus (Qiagen, Valencia, CA) for maximal recovery of high quality RNA or PBS for immediate cytospin preparation. To detect BCR-ABL1 by fluorescence *in situ* hybridization, the Vysis LSI BCR/ABL Dual Color Dual Fusion Translocation Probe (Abbott Laboratories, Abbott Park, Illinois, USA) was used. Fluorescent signals were visualized using an Axioskop 2 mot *plus* with an AxioCam microscope camera (Carl Zeiss Microscopy, LLC, Thornwood, NY, USA).

### RNA extraction and gene expression profiling

Total RNA and gDNA were purified from randomized lysates with the AllPrep kit (Qiagen Valencia, CA). cDNA synthesis, amplification and labeling were performed with the Ovation Pico WTA System vV2 and Encore Biotin (NuGEN Technologies, San Carlos, CA) with 2 ng of total RNA. Labeled hybridization targets were hybridized to GeneChip HumanGenome-U133 Plus 2.0 microarrays (Affymetrix, Santa Clara, CA). Nucleic acid extractions and microarray assays were performed at Oregon Health & Science University (OHSU Gene Profiling Shared Resource).

### Microarray data analysis

#### Quality control of samples

Samples were background-corrected and normalized with *gcrma*. All 38 samples passed quality controls for 5′ to 3′ RNA degradation rates. Three samples exhibited large probe-set heterogeneity based on relative log expression and Normalized Unscaled Standard Error diagnostic plots and were removed from downstream analysis.

#### Microarray data analysis

All 54,675 Affymetrix probes were evaluated for differential expression between responders (defined as DMR at ≥1 evaluation) and non-responders by applying R software package *limma*. Sparse principal component analysis was applied to the microarray using the arrayspc function from the elasticnet R package with default parameters.

Probe expression for heat maps was reported as probe-wise z-scores of the normalized expression values, where probes with no variation across samples were assigned a zero. Heat maps arrange the normalized probe-level expression by row and patients by column. Genes for the 75-probe signature predictive of MCyR in CP-CML patients treated with imatinib were obtained from McWeeney *et al.* [8]. Genes implicated in CML leukemia stem cell (LSC) persistence were obtained via literature review. Genes in the Wnt/β-catenin signaling pathway (KEGG ID: hsa04310) were obtained through Bioconductor’s KEGGREST API. There was a median of 2 probes per gene for the LSC gene set, where probes are annotated at the gene level. Probes of the Wnt/β-catenin signaling heat map are annotated according to genes and/or gene families with at least 8 probes mapping to a gene or gene family. Probes in the Wnt signalling pathway that had <8 probes per gene or gene family were labeled as “other”. The median unique genes per annotated group was 3, while the median number of probes per annotated group was 11.

### Statistics

Baseline parameters between responders and non-responders were assessed using Mann-Whitney *U* tests with a family-wise error rate of 0.05.

## SUPPLEMENTARY MATERIALS FIGURES


