# Supplementary material for: Similar expression profiles in CD34+ cells from chronic phase chronic myeloid leukemia patients with and without deep molecular responses to nilotinib
Source: Oncotarget. 2018 Apr 3;9(25):17889–94. doi: 10.18632/oncotarget.24954 (PMC5915162; doi:10.18632/oncotarget.24954)
Supplement: Supplementary file 1 [file oncotarget-09-17889-s001.pdf]

Similar expression profiles in CD34$^+$ cells from chronic phase chronic myeloid leukemia patients with and without deep molecular responses to nilotinib

SUPPLEMENTARY MATERIALS

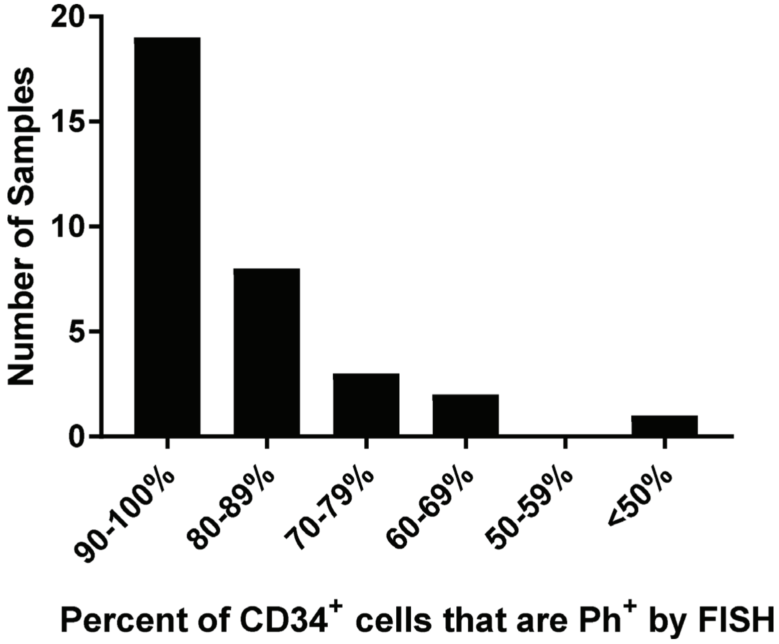

Supplementary Figure 1: Graph showing the percentage of CD34+ cells that were BCR-ABL1 positive by FISH (X-axis) and the number of samples which fell into each percentage category (Y-axis).

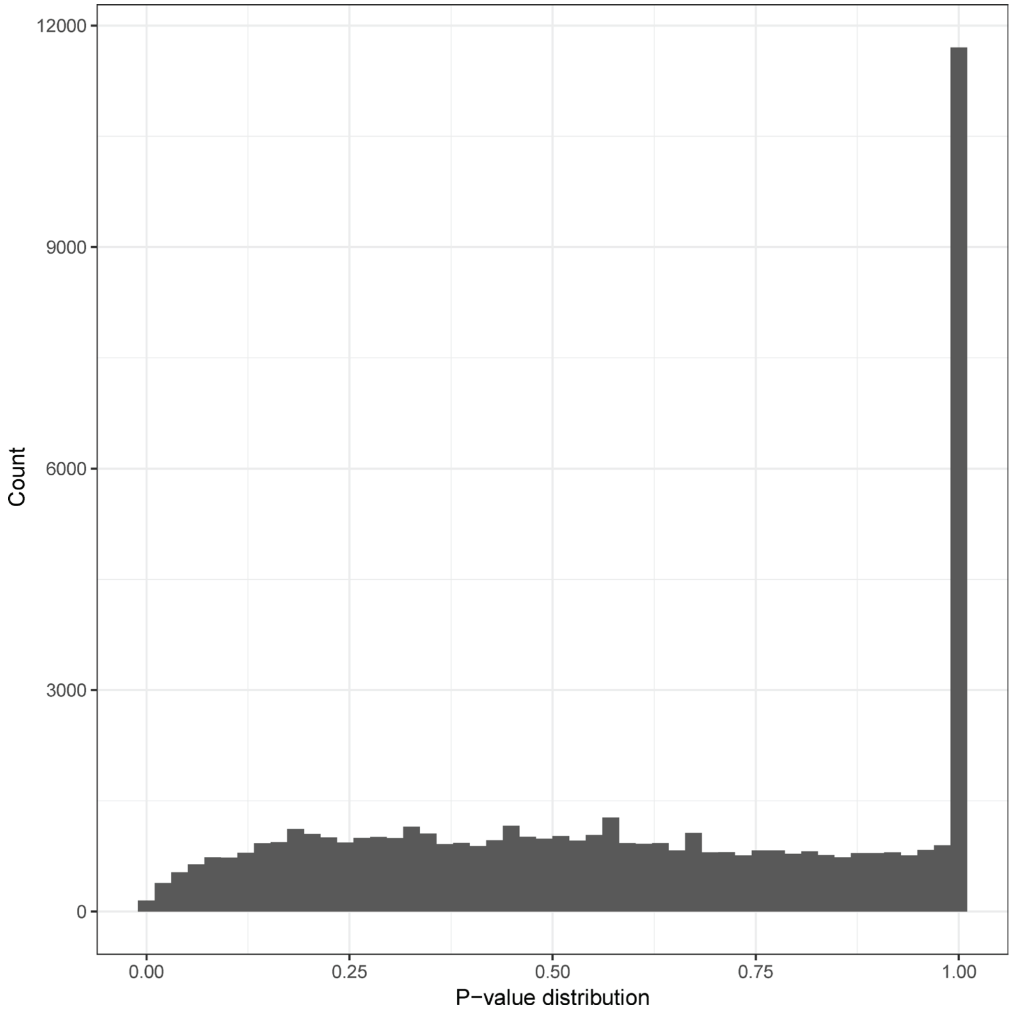

Supplementary Figure 2: Distribution of *p*-values for the 54,675 microarray probes following application of *limma*.

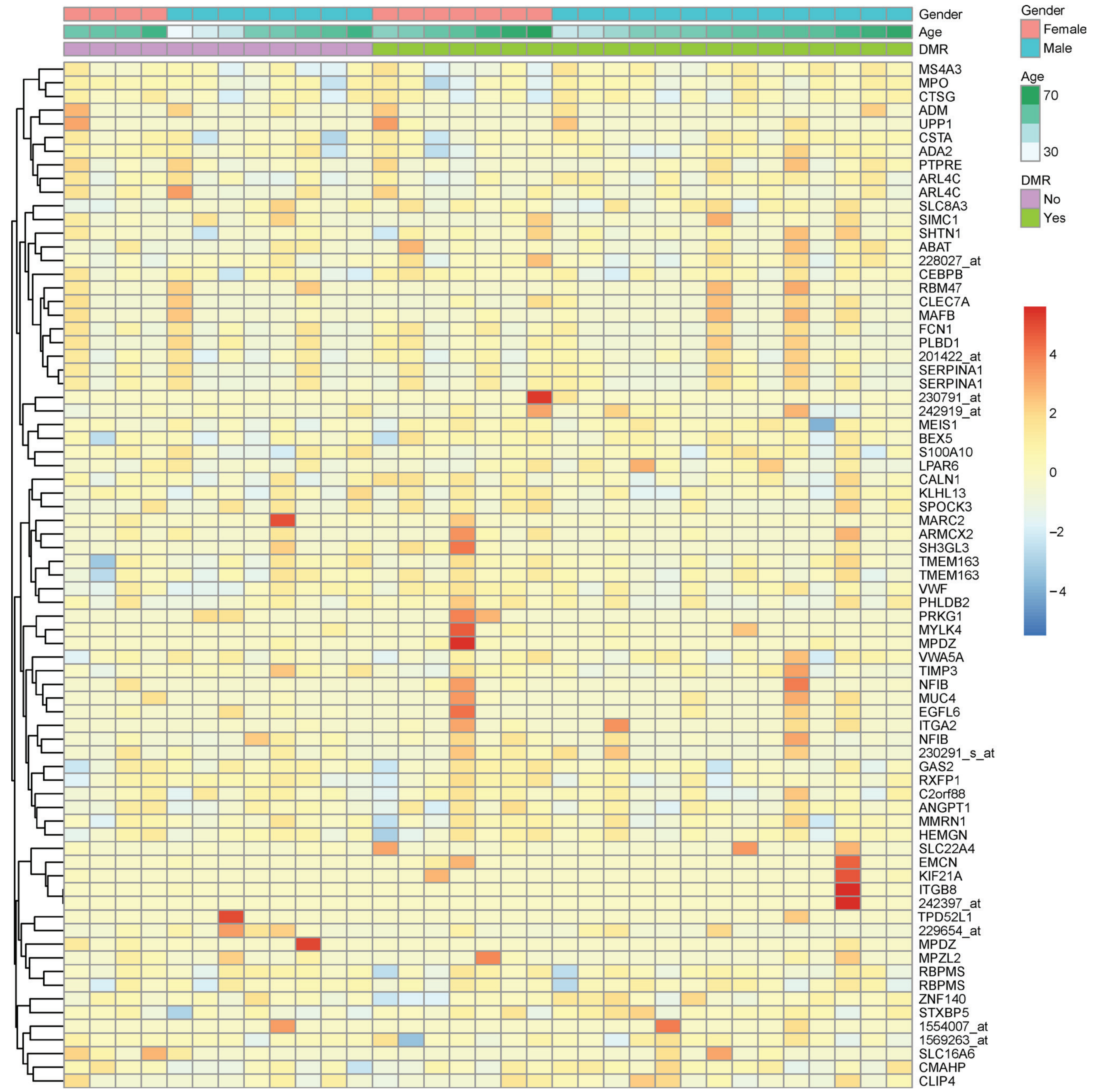

Supplementary Figure 3: Heat map showing the expression of 75 probes identified by McWeeney *et al*. [8] in patients with and without DMR on nilotinib.

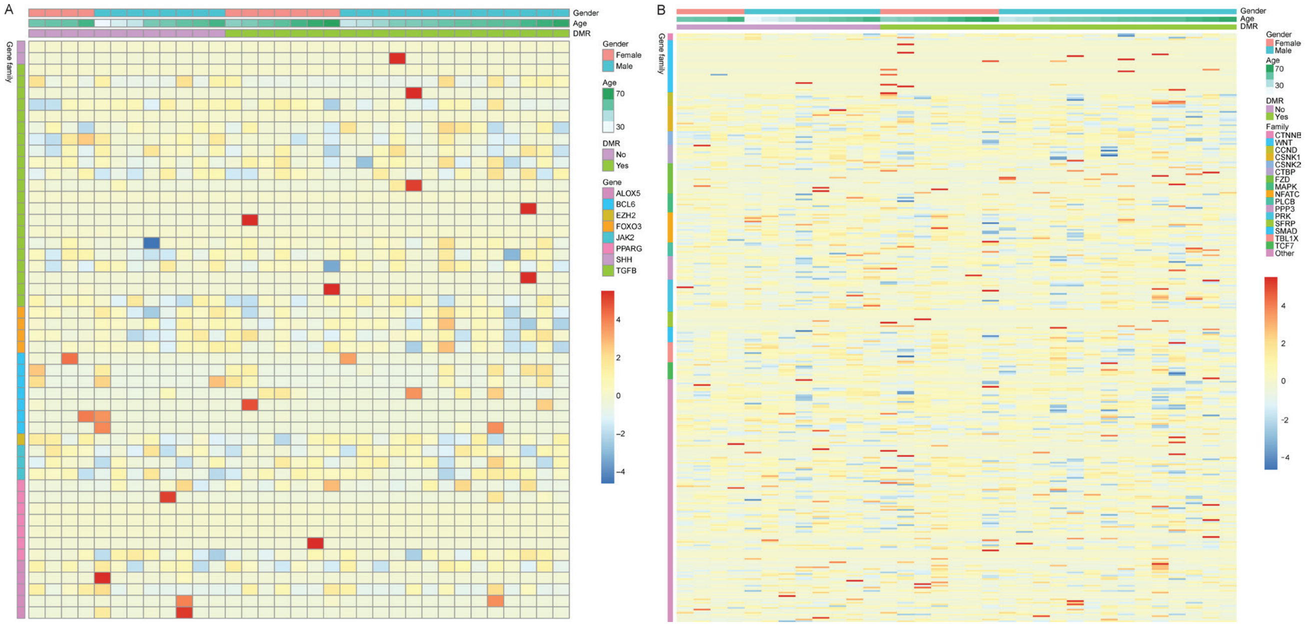

Supplementary Figure 4: (**A**) Heat map showing the expression of 50 probes representing genes associated with leukemia persistence patients in patients with and without DMR on nilotinib. (**B**). Heat map showing the expression of 365 probes associated with Wnt/β -catenin signaling in patients with and without DMR on nilotinib.
